# Multiple Imputation of Missing Complex Survey Data using SAS^®^: A Brief Overview and An Example Based on the Research and Development Survey (RANDS)

**Published:** 2023-01

**Authors:** Yulei He, Guangyu Zhang

**Affiliations:** 1 Division of Research and Methodology, U.S. Centers for Desease Control and Prevention; 2 National Center for Health Statistics, U.S. Centers for Desease Control and Prevention

**Keywords:** Complex Survey, Missing Data, Multiple Imputation, SAS^®^

## Abstract

Multiple imputation (MI) is a widely used analytic approach to address missing data problems. SAS^®^ (SAS Institute Inc, Cary, N.C.) has established MI procedures including PROC MI and PROC MIANALYZE. We illustrate the use of these procedures for conducting MI analysis of complex survey data by an example from RANDS. Section 1 contains the introduction. Section 2 includes some necessary methodological background. Section 3 shows a MI example with an arbitrary missing data pattern. Section 4 concludes the paper with a discussion.

## 1 Introduction

Population-based studies often rely on surveys to collect information and conduct data analysis. However, survey data are often subject to nonresponse or missing data problems. Multiple imputation (MI) is arguably one of the most popular statistical strategies to handle missing data issues in many fields (Rubin 1987; He et al., 2022) including survey nonresponse problems.

The default option in statistical software is to remove cases with missing values from the analysis (i.e., case-deletion). The practicality of MI sits on its successful implementations in some mainstream software packages (e.g., SAS^®^ and R) so that practitioners can use straightforward programming statements to conduct the analysis. For example, Berglund and Heeringa (2014) provided an overview of MI and its applications, using SAS^®^ for illustration. Similar research literature can be found for other software packages. In addition, practitioners can refer to the software documentation for guidance.

Missing data problems in complex surveys pose some unique challenges (Section 2). For survey item nonresponse problems, MI has been proven to be a useful analytical tool supported by a large body of literature (e.g., Rubin 1987; He et al. 2022, Chapter. 10). However, most of the literature has focused on the technical aspects of MI and yet touched less on the programming components. In addition, the relevant programming literature and documentation are largely targeted to non-survey types of data.

To fill this gap, the aim of this paper is to provide a brief overview and a real example of MI for complex survey data using SAS^®^ programming statements (version 9.4; additionally, the users can also use the free cloud SAS platform on https://www.sas.com/en_us/software/on-demand-for-academics.html).

## 2. Method Background

### 2.1 Missing data mechanism

Briefly speaking, the missing data mechanism of an incomplete variable describes how the probability of its missingness (i.e., being missing) is related to the original data. In general, there are three types of missing data mechanisms: (1) Missing completely at random (MCAR): the missingness of a variable is not related to any variable in the data; (2) Missing at random (MAR): the missingness of a variable is only related to other fully-observed variables in the data; (3) Missing not at random (MNAR): the missingness of a variable is related to the missing values after controlling for other fully-observed variables.

### 2.2 Multiple Imputation

To conduct a MI analysis of a dataset, an appropriate missing data mechanism (e.g., MAR) is first assumed. Then a statistical imputation model is formulated to relate the missing variable(s) with observed variable(s) in the dataset. Next, missing values are imputed (i.e., replaced) by random draws from their posterior predictive distributions or their approximations derived from the imputation model. Such a procedure is independently repeated multiple (say M) times, resulting in M sets of imputed values. Early research (e.g., Rubin 1987) suggested setting M=5 is sufficient for regular analyses applied to datasets with a small or moderate amount of missing data. More recent research (e.g., He et al. 2022, Section 3.3.3) has shown that larger numbers (e.g., M > 5) might be desired when computing and data storage resources are available. After imputation, each of the M completed datasets, including both the observed and the imputed values, is analyzed separately and results in M sets of analysis results/estimates. Finally, these M sets of results are combined to yield a single set of statistical inference using the so-called Rubin’s combining rules (Rubin 1987).

### 2.3 Multiple Imputation for Complex Survey Missing Data Problems

Most surveys are based on sample designs with one or more complex features such as stratification, clustering of sampled elements, and weighting to compensate for differential probabilities of sample inclusion or varying response rates. Therefore, it is essential to incorporate this design information for survey data analysis (Cochran 1977). Survey data analysis procedures accounting for the design information are readily available in SAS^®^ (Section 3).

The above principle also holds for analyzing multiply-imputed complex survey data. Additionally, a principled MI procedure for complex survey missing data problems should also include the design information in the imputation process. However, there exist alternative practical options for incorporating the sample design (e.g., He et al. 2022, Section 10.3). Here we outline a hierarchical, trial-and-error strategy:

1. Include the survey weight as a variable (predictor) in the imputation;
2. To include information about the sampling strata and clusters:
  (2.1) First, create a new categorical variable that combines the sampling strata and the nested clusters, and include this variable in the imputation;
  (2.2) If the imputation model has some estimation issues due to a large number of categories from the above combining variable, then collapse clusters within a sampling stratum for clusters with small sample sizes or only includes the sampling strata variable in the imputation;
  (2.3) If the model estimation issue still exists because some strata only have very few units then collapse these small-sample strata together to ensure each final stratum has a sufficient sample size, and then include the collapsed-strata variable in the imputation.

An additional major challenge for surveys is that missing data often happen for multiple variables, and this issue is usually coupled with another fact that survey variables are typically bounded. A feasible MI approach is the so-called “Fully Conditional Specification” (FCS) strategy, which imputes each incomplete variable based on a model that includes all other variables as the predictors and then cycles through all missing variables sequentially. FCS is arguably the most popular MI strategy for multivariate survey missing data problems (He et al. 2022, Chapter. 7).

## 3. A Multiple Imputation Example using SAS^®^

### 3.1 Major SAS^®^ Procedures

The two main SAS^®^ procedures for MI are PROC MI and PROC MIANALYZE. Other SAS^®^ procedures and data steps are also often used, depending on the analytic goals and contexts. Here we outline five major programming stages in a typical MI analysis.

Stage 1 (processing): Processing data before imputation to construct the working dataset including both the target missing and fully-observed variables. Exploratory analyses are often conducted at this stage.

Stage 2 (imputation): Running imputation M times by applying PROC MI to the working dataset.

Stage 3 (analysis): Applying the planned (post-imputation) analysis to the completed datasets by running SAS^®^ statistical procedures. In the context of complex survey data, these procedures typically include PROC SURVEYMEANS, PROC SURVEYREG, etc.

Stage 4 (combining): Combining the results to yield the final estimates with PROC MIANALYZE.

Stage 5 (evaluation): An evaluation analysis that typically compares results among different MI models and with the case-wise deletion method.

### 3.2 Data Background

The example is illustrated using a subset of Research and Development Survey (RANDS) (https://www.cdc.gov/nchs/rands/), a series of probability-sampled web-based surveys conducted by the National Center for Health Statistics (e.g., He et al, 2020). Specifically, we use some variables from the publicly released RANDS during COVID-19 data (the 3^rd^ round), which is a special series of RANDS used to rapidly report on the impact of the COVID-19 pandemic (Irimata and Scanlon, 2022). The original dataset contains 5,458 records; it can be downloaded from (https://www.cdc.gov/nchs/rands/data.htm). Table 1 briefly describes the variables used in the example.

### 3.3 Sample Code and Output

Stage 1: The selected variables contain no missing values in the original data. For illustrative purpose, we created around 20% missing values in both INCOME and MARITAL_NEW. The missingness of INCOME is related to AGE, GENDER, EDUC, and INTERNET; the missingness of MARITAL_NEW is related to AGE, EDUC, INTERNET, and HHSIZE. The missingness of both variables follows MAR (Section 2.1). For illustration, the key missing data-generating step for INCOME is included as follows (the initial dataset is called rands_covid3_new, while the new one is called rands_covid_missing):


data rands_covid_missing; 
  set rands_covid3_new; 
  p_miss_INCOME = exp(−2+0.5*EDUC−0.5*GENDER−0.01*AGE+0.5*INTERNET) 
          /(1+exp(− 2+0.5*EDUC−0.5*GENDER−0.01*AGE+0.5*INTERNET)); 
  rnumber_INCOME = ranuni(20110411); 
  If rnumber_INCOME < p_miss_INCOME then R_miss_INCOME =1; 
           else R_miss_INCOME=0; 
  If R_miss_INCOME = 1 then INCOME=.; 
run; 


In SAS^®^, missing values are coded by “.” (dot). In the code above, INCOME is set as missing if a uniform random number is less than a pre-specified missingness probability, which is related to other variables by a logit function. As outlined in Section 3.1, additional SAS^®^ data steps and exploratory analyses can be done for the data processing stage of the MI analysis.

Stage 2: We first briefly discuss some possible modeling strategies. Since both INCOME and MARITAL_NEW have missing values, the desirable imputation strategy is FCS (Section 2.3). Under FCS, there exist alternative modeling options, some of which are included as follows:

1. INCOME has 16 categories (i.e., 1–16) with an ordinal nature. Although each integer value does not represent the same dollar amount range, for simplicity we only consider these integers as our imputation and analysis metric. For convenience of illustration, this variable can be treated as a positive continuous variable and modeled via a linear regression model conditional on other variables. However, the imputed INCOME values can take fractional numbers. To preserve the integer format, a naive post-imputation rounding step can be taken; imputed values less than 1 can be set as 1 and those above 16 can be set as 16. Additionally, PROC MI has an option to force the imputed values being generated within a pre-specified range (e.g., [1,16]), and then rounding is only necessary for imputed values within the range. On the other hand, INCOME can also be imputed using the predictive mean matching (PMM) method (e.g., He et al. 2022, Section 5.5). Briefly, PMM can be viewed as a MI extension of hot-deck imputation, where each missing value is replaced with an observed response from a “similar” unit. In our example, PMM can naturally preserve the range and integer format of the imputations without the need of rounding.
2. ARITAL_NEW has two categories, it can be modelled using a logistic regression conditional on other variables. Alternatively, binary or nominal variables such as MARITAL_NEW can be imputed via a discriminant analysis model. That is, stratified by MARITAL_NEW, other variables are assumed to follow a multivariate normal distribution (e.g., He et al. 2022, Section 4.3.2).

The sample code is as follows:


proc mi data =rands_covid_missing seed =197789 out= income_impute nimpute =5 
               min = 1 . . . . . . . . max = 16 . . . . . . . . ; 
        class EDUC GENDER INTERNET MARITAL_NEW S_VSTRAT_COMBINE ; 
       fcs nbiter=20 reg (INCOME/details) logistic (MARITAL_NEW / details likelihood=augment) ; 
       *fcs nbiter=20 regpmm (INCOME/details) logistic (MARITAL_NEW/details likelihood=augment); 
       *fcs nbiter=20 reg (INCOME/details) discrim (MARITAL_NEW/classeffects =include details); 
       *fcs nbiter=20 regpmm (INCOME/details) discrim (MARITAL_NEW/classeffects=include details); 
       var INCOME AGE WEIGHT_CALIBRATED EDUC GENDER INTERNET MARITAL_NEW HHSIZE S_VSTRAT_COMBINE; 
run; 


We provide some additional remarks about the above code.

- The input dataset is “rands_covid_missing”; the output dataset containing the multiple imputation results is “income_impute”; “nimpute=” specifies the number of imputations (we use 5 in this example); “seed=” specifies the initial random seed used in MI. Fixing the random seed can render reproducible results.
- The variables included in the imputation are specified after “var”. Among them, categorical variables are specified after “class”.
- To include the design variables, we initially include WEIGHT_CALIBRATED and the combined strata and PSU variable (S_VSTRAT and S_VPSU, respectively) in the model (after “var”). However, the model has estimation problems because some sampling strata have very few samples. As a result, SAS^®^ would issue warnings in log files. They would also be noticed by checking the regression coefficients of the output. Therefore, we collapse some small strata so that each final stratum has at least 10 samples, which is coded by the new variable S_VSTRAT_COMBINE. We also exclude S_VPSU from the model.
- fcs nbiter=20 reg (INCOME/details) logistic (MARITAL_NEW/details likelihood=augment). This statement specifies that we use FCS to impute both INCOME and MARITAL_NEW. Specifically, “nbiter=20” specifies 20 iterations are to be used; “reg (INCOME/details)” specifies a linear regression model for INCOME, and the “details” option asks for outputting the regression coefficients of the model fit across all imputations; “logistic/details” specifies a logistic regression imputation model for MARITAL_NEW with coefficients output; “likelihood=augment” specifies a robust logistic regression to deal with possible data separation issues (e.g., He et al. 2022, Section 4.3.2.4).
- We can specify “min=1” and “max=16” after “proc” to force the imputed values of INCOME falling in this range. For the variables that do not need the bounds, their “min” and “max” are assigned as missing values.
- fcs nbiter=20 regpmm (INCOME/details) logistic (MARITAL_NEW/details likelihood=augment).
  This statement (commented out with a “*”) specifies another modeling option: a PMM imputation for INCOME and a logistic regression imputation for MARITAL_NEW.
- fcs nbiter=20 reg (INCOME/details) discrim (MARITAL_NEW/classeffects =include details). This statement (commented out with a “*”) specifies another modeling option: a linear normal imputation for INCOME and a discriminant analysis model for MARITAL_NEW. For the latter, “classeffects=include” specifies that all of the remaining variables, both continuous and categorical, are included in the discriminant analysis.
- fcs nbiter=20 regpmm (INCOME/details) discrim (MARITAL_NEW/classeffects =include details). This statement (commented out with a “*”) specifies another modeling option: a PMM imputation for INCOME and a discriminant analysis model for MARITAL_NEW.

We now include some output from the above code and provide remarks. For ease of illustration, we separate the output into four parts and then comment on them one by one.

Output 1

**Table:** 

The MI Procedure	
Model Information	
**Data Set**	WORK.RANDS_COVID_MISSING
**Method**	FCS
**Number of Imputations**	5
**Number of Burn-in Iterations**	20
**Seed for random number generator**	197789
FCS Model Specification	
Method	Imputed Variables
Regression	INCOME AGE WEIGHT_CALIBRATED HHSIZE
Logistic Regression	MARITAL_NEW
Discriminant Function	EDUC GENDER INTERNET S_VSTRAT_COMBINE

Output 1 provides some general information about the imputation model setup and the variables included. For categorical variables, the discriminant analysis imputation model is the default option.

Output 2 shows the missingness pattern of the variables and some descriptive statistics of the associated subgroups. Specifically, Group 1 has all variables fully observed, denoted by ‘X” for each variable; Group 2 has only MARITAL_NEW with missing values (denoted by “.”); Group 3 has only INCOME with missing values; and Group 4 has missing values on both INCOME and MARITAL_NEW. The means of the continuous variables of each subgroup are also displayed. For instance, the average age from Group 1 (=53.386) is higher than those from the other three groups.

Output 2

**Table:** 

Missing Data Patterns															
Group	INCOME	AGE	WEIGHT_CALIBRATED	EDUC	GENDER	INTERNET	MARITAL_NEW	HHSIZE	S_VSTRAT_COMBINE	Freq	Percent	Group Means			
												INCOME	AGE	WEIGHT_CALIBRATED	HHSIZE
**1**	X	X	X	X	X	X	X	X	X	3289	60.26	9.9811	53.386	0.9476	2.4387
**2**	X	X	X	X	X	X		X	X	948	17.37	9.9535	48.800	1.1708	3.9409
**3**		X	X	X	X	X	X	X	X	941	17.24		49.865	0.9972	2.5622
**4**		X	X	X	X	X		X	X	280	5.13		46.867	1.0469	4.2107

Output 2 also shows that the data have an arbitrary missing data pattern. On the opposite, a monotone missingness pattern is usually seen in longitudinal studies where once a subject drops out, his/her measurements at later times are always missing. Note that PROC MI has specific options for imputing monotone missing data. However, for brevity, they are not covered in this paper.

Output 3

**Table:** 

Regression Models for FCS Method							
Imputed Variable	Effect	EDUC	Imputation				
			1	2	3	4	5
**INCOME**	**Intercept**	.	−0.223674	−0.202220	−0.219967	−0.191550	−0.188843
**INCOME**	**AGE**	.	0.020476	0.029064	0.038018	0.022661	0.018259
**INCOME**	**WEIGHT_CALIBRATED**	.	0.042331	0.031441	0.069224	0.061784	0.034479
**INCOME**	**EDUC**	2.000	−0.377725	−0.396039	−0.394906	−0.384208	−0.329835
**INCOME**	**EDUC**	3.000	−0.036021	−0.014313	−0.023572	−0.011021	−0.077057

Logistic Models for FCS Method						
Imputed Variable	Effect	Imputation				
		1	2	3	4	5
**MARITAL_NEW**	**Intercept**	−0.246513	−0.105072	−0.137450	−0.165294	−0.110486
**MARITAL_NEW**	**INCOME**	−0.885190	−0.903693	−0.923058	−0.902241	−0.809001
**MARITAL_NEW**	**AGE**	−0.524000	−0.520803	−0.542015	−0.571203	−0.524950
**MARITAL_NEW**	**WEIGHT_CALIBRATED**	−0.339081	−0.282057	−0.328680	−0.264126	−0.374018

Output 3 shows some details about the fit for each of the imputation models used in FCS. If we use the modeling option “fcs nbiter=20 reg (INCOME/details) logistic (MARITAL_NEW/details likelihood=augment)” in PROC MI, then the output contains the linear regression coefficients for INCOME and logistic regression coefficients for MARITAL_NEW across 5 imputations. For simplicity we do not include all coefficients here. Specifically, the results under “Regression Models for FCS Method” lists the coefficients for fitting INCOME. For example, the coefficient for AGE is 0.020476 for the 1^st^ imputation, 0.029064 for the 2^nd^ imputation, etc. The results under “Logistic Models for FCS Method” lists the coefficients for fitting MARITAL_NEW. For instance, the coefficient for AGE is −0.524000 for the 1^st^ imputation, −0.520803 for the 2^nd^ imputation, etc.

We previously discussed the need for collapsing some small strata and excluding clusters to achieve stable model estimates. If this was not implemented, in addition to seeing warning statements from SAS^®^ log files, we would also see some very extreme logistic regression coefficients (e.g., outside the range [−5,5]) in Output 3.

Output 4

**Table:** 

Variance Information (5 Imputations)							
Variable	Variance			DF	Relative Increase in Variance	Fraction Missing Information	Relative Efficiency
	Between	Within	Total				
**INCOME**	0.001115	0.003021	0.004360	41.96	0.443073	0.337540	0.936761

Parameter Estimates (5 Imputations)										
Variable	Mean	Std Error	95% Confidence Limits		DF	Minimum	Maximum	Mu0	t for H0: Mean=Mu0	Pr > |t|
**INCOME**	10.014665	0.066028	9.881411	10.14792	41.96	9.974504	10.065734	0	151.67	<.0001

Output 4 shows some combined estimates after MI. It only displays simple means for continuous variables (e.g., INCOME) and some associated statistics. Note that it might be inappropriate to use this output as the basis for final results. For example, the mean estimation of INCOME here does not account for the complex survey design of RANDS.

Stage 3: we use the mean estimates as an analytical example. The example code is as follows:


proc surveymeans data=income_impute; 
   weight WEIGHT_CALIBRATED; 
   strata S_VSTRAT; 
   cluster S_VPSU;
   var INCOME MARITAL_NEW; 
   by _imputation_; 
   ods output Statistics = mean_income_imp; 
run; 


For illustration, we estimate the overall mean of INCOME and MARITAL_NEW using PROC SURVEYMEANS, which uses the survey design information including strata, clusters, and weights. The working dataset “data=income_impute” reads the output dataset from PROC MI. In that dataset, a variable “_imputation_” is used to label the number of imputations (i.e., 1–5), and the dataset has 27,290 (=5458×5) records. A “by” option is used to run the analyses separately. Finally, the “ods output statistics = mean_income_imp” is used to store the output of the 5 analyses in the dataset “mean_income_imp” for carrying out the combining step in Stage 4.

Output 5 shows the means and standard errors of both variables from the 1^st^ imputed dataset. It contains the default output from PROC SURVEYMEANS. For example, the mean of the completed INCOME is 10.161342 and the standard error estimate is 0.110726. The full SAS^®^ output would include results from all 5 imputations and distribution plots of both variables (details not shown).

Output 5

**Table:** 

The SURVEYMEANS Procedure Imputation Number=1	
Data Summary	
**Number of Strata**	71
**Number of Clusters**	159
**Number of Observations**	5458
**Sum of Weights**	5457.99708

Statistics					
Variable	N	Mean	Std Error of Mean	95% CL for Mean	
**INCOME**	5458	10.161342	0.110726	9.94129666	10.3813876
**MARITAL_NEW**	5458	0.646931	0.010449	0.62616660	0.6676959

Stage 4: We synthesize the results from the multiply-imputed datasets using PROC MIANALYZE.

For example, the following code combines the survey mean estimates for INCOME.


proc mianalyze data =mean_income_imp edf=88; 
   modeleffects mean; 
   stderr stderr; 
   where varname = ‘INCOME’; 
   ods output parameterestimates=MI_results_income; 
run; 


The procedure reads in the dataset mean_income_imp, which contains the separate estimates from the multiply-imputed datasets. The option “EDF= ” is not the default but necessary for complex survey data analysis because it specifies the degrees of freedom in the combining step. In this example, we specify the degrees of freedom as the number of clusters minus the number of strata in the dataset. The statement “modeleffects mean” specifies that the estimand for combining is the mean estimates. The statement “stderr stderr” lists standard errors associated with the means. “where varname = ‘INCOME’” indicates that the combining step only applies to INCOME. Finally, “ods output parameterestimates=MI_results_income” saves the combined estimates to the dataset MI_results_income.

Output 6

**Table:** 

The MIANALYZE Procedure	
Model Information	
**Data Set**	WORK.MEAN_INCOME_IMP
**Number of Imputations**	5

Variance Information (5 Imputations)							
Parameter	Variance			DF	Relative Increase in Variance	Fraction Missing Information	Relative Efficiency
	Between	Within	Total				
**Mean**	0.000756	0.013659	0.014453	80.302	0.058117	0.055225	0.997246

Parameter Estimates (5 Imputations)										
Parameter	Estimate	Std Error	95% Confidence Limits		DF	Minimum	Maximum	Theta0	t for H0: Parameter=Theta0	Pr > |t|
**Mean**	10.230448	0.120219	9.991217	10.46968	80.302	10.196713	10.294728	0	85.10	<.0001

Output 6 shows the results from PROC MIANALYZE. The combined mean estimate of INCOME is 10.230448, its standard error is 0.120219, and the 95% confidence limits are (9.991212, 10.46968). Detailed explanations of other statistics (e.g., between/within variance) can be found in the literature (e.g., He et al. 2022, Chapter. 3).

Stage 5: We conduct some diagnostics and evaluation. We have considered different modeling options for INCOME and MARITAL_NEW (Section 3.2.2). In this example, since we create the missing values, the imputation analysis results can also be compared with those from complete data as well as from the case-deletion method. The programming code for Stage 5 would be running different MI models and analyses (e.g., remark (d)-(h) after PROC MI in Section 3.3). Omitting the details, the evaluation results are summarized in Table 2.

The mean estimates from the case-deletion are considerably lower than the complete-data analysis due to MAR. In general, all MI methods correct for the biases somewhat. In addition, MI analyses yield generally narrower confidence intervals than the case-deletion method. Among different MI methods applied, it seems that when INCOME is imputed via PMM, the corresponding results are the closest to the complete-data analysis for both variables. Therefore, we would choose PMM+logit as the final MI modeling option.

## 4. Discussion

We provide some simple illustrations on how to use SAS^®^ to conduct MI analysis for complex survey data. In addition to providing some sample code and output, we provide some general guidance on constructing imputation models and running some evaluations. The full programming code is available at https://github.com/he-zhang-hsu/multiple_imputation_book/tree/Survey_statistician. Additional references on SAS^®^-based MI applications can be found in Berglund and Heeringa (2014) and relevant SAS documentation. References on MI strategies and applications, including non-survey data and how they can be implemented using other software packages such as R (https://www.R-project.org/) package “mice” (see van Buuren and Groothuis-Oudshoorn, 2011), can be found in He et al. (2022).

## Figures and Tables

**Table 1:** Variables Used in the Example

Variable	SAS^®^ name	Specifications
Age in years	AGE	18–70; Age ≥ 70 is top-coded
Sex	GENDER	Male/Female
Education	EDUC	High school diploma or less/ Some college/Bachelor’s degree or higher
Marital status	MARITAL_NEW	Married or living with partners / Others*
Household internet use	INTERNET	Yes/No
Household size	HHSIZE	1–6; household size >=6 is top-coded
Household income	INCOME	1–16**
Sampling strata	S_VSTRAT	71 sampling strata in the original data
Sampling clusters	S_VPSU	2 to 7 clusters per stratum
Survey weights	WEIGHT_CALIBRATED	0.0096–17.6472***

Note:

* collapsed from 6 categories in the original data; “Others” has four categories: widowed, divorced, separated, and never married.

** 1: < $5000; 2: $5000–9999; 3: $10000–14999; 4: $15000–19999; 5: $20000–24999; 6: $25000–29999; 7: $30000–34999; 8: $35000–39999; 9: $40000–49999; 10: $50000–59999; 11: $60000–74999; 12: $75000–84999; 13: $85000–99999; 14: $100000–124999; 15: $125000–149999; 16: > $150000.

*** normalized survey weights after calibrating to adjust for possible selection bias of RANDS.

**Table 2:** Mean Estimates of INCOME and MARITAL_NEW from Different Methods

Method	INCOME	MARITAL_NEW
Complete-data	10.38 (10.14, 10.62)	0.613 (0.592, 0.634)
Case-deletion	10.17 (9.91, 10.43)	0.589 (0.565, 0.614)
MI: linear+logit	10.36 (10.12, 10.59)	0.624 (0.600, 0.648)
MI: linear+discriminant	10.35 (10.13, 10.58)	0.620 (0.596, 0.643)
MI: (constrained) linear+logit	10.26 (10.03, 10.49)	0.621 (0.597, 0.644)
MI: (constrained) linear+discriminant	10.26 (10.03, 10.49)	0.623 (0.600, 0.645)
MI: PMM + logit	10.39 (10.17, 10.62)	0.621 (0.597, 0.644)
MI: PMM + discriminant	10.40 (10.16, 10.64)	0.620 (0.596, 0.644)

Note: 1. 95% confidence intervals are in the parentheses. 2. INCOME is modelled by either “linear” or “PMM”; MARITAL_NEW is modelled by either “logit” or “discriminant”. 3. “constrained” denotes imputed values for INCOME are forced to be in [1,16]. 4. Rounding is applied for fractional numbers when applicable.

## References

[R1] Berglund P and Heeringa S. Multiple Imputation of Missing Data Using SAS. 2014. Cary, NC: SAS Institute Inc.

[R2] Cochran WG. Sampling Techniques, 3rd Edition. 1977. New York: Wiley.

[R3] He Y, Cai B, Shin H-C, Beresovsky V, Parsons V, Irimata K, The National Center for Health Statistics’ 2015 and 2016 Research and Development Surveys. National Center for Health Statistics. Vital Health Stat 1(64). 2020. 33151143

[R4] He Y, Zhang G, Hsu CH. Multiple Imputation of Missing Data in Practice: Basic Theory and Analysis Strategies, 1st Edition. 2022. Chapman and Hall/CRC Press.

[R5] Irimata KE and Scanlon PJ. 2022. The Research and Development Survey (RANDS) during COVID-19. Statistical Journal of the International Association for Official Statistics 38(1): 13–21. 35928170 10.3233/SJI-210880 PMC9345600

[R6] Rubin DB. Multiple Imputation for Nonresponse in Surveys. 1987. New York: Wiley.

[R7] van Buuren S and Groothuis-Oudshoorn K (2011). mice: Multivariate Imputation by Chained Equations in R. Journal of Statistical Software, 45(3), 1–67. DOI 10.18637/jss.v045.i03.

